# Tongue and groove (v-shaped) osteotomy in treatment of non-union of long bones - a novel surgical technique

**DOI:** 10.1308/003588412X13171221591259j

**Published:** 2012-05

**Authors:** S Mukhopadhyay, N Vannet, S Morgan-Jones

**Affiliations:** Department of Orthopaedics, University Hospital of WalesUK

## BACKGROUND

**Figure 1:**
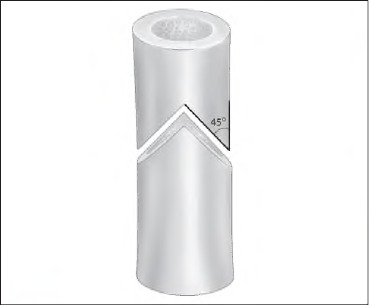
Tongue and groove osteotomy

To treat long-bone non-unions it is important to create a stable mechanical environment in which fracture healing can take place securely. Interfaces such as transverse or step-cut osteotomy can still be improved. The method of tongue and groove osteotomy (Figure 1) described here is a viable option in difficult non-union surgery.

## METHODS

Four patients treated for non-union of humerus (closed injury) and one for non-union of femur fracture (open injury leading to below-knee amputation) were followed up for 5 years (2003–2008).

**Figure 2:**
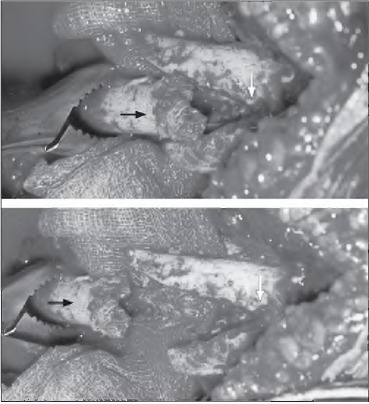
Intraoperative picture showing creation of tongue in groove (black arrow: tongue; white arrow: groove)

**Figure 3:**
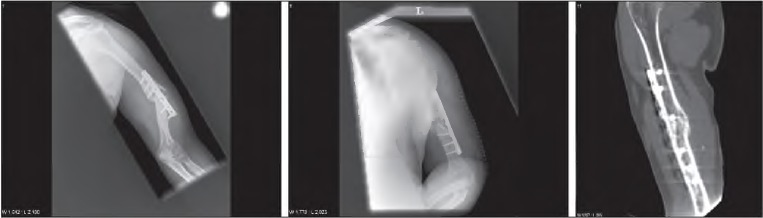
Pre and post-operative images with evidence of healing - humeral non-union

**Figure 4:**
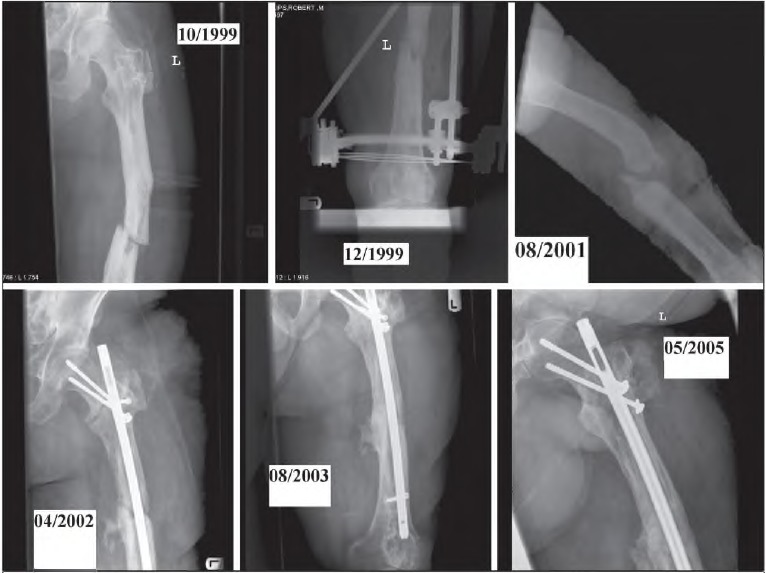
Pre and post-operative images with evidence of healing - femoral non-union

After the fracture site was exposed and freshened, a 45-degree (Figure 1) tongue-shaped tower end was created at the proximal end of the distal fragment using an oscillating saw (Figure 2). On the distal part of the proximal end a corresponding ‘groove’ was made. Dynamic compression plating for humerus and intramedullary nailing for femur was used (Figures 3 and 4) with appropriate early postoperative mobilisation.

## DISCUSSION

Humeral non-unions united between four and six months and the femoral non-union united in six months. Radiological signs of union were achieved earlier in each case than clinical union. Long-term follow-up showed satisfactory result in all cases.

In a study conducted with frozen porcine femur model it was observed that v-shaped osteotomy with a docking angle between 45 and 90 degrees can withstand a strong compression load.^1^ The tongue-and-groove osteotomy in long-bone non-union surgery is better than step cut osteotomy because of increased contact surface area, improved rotational stability^2^,^4^ and easy reproducibility. Clinical results of similar surgical procedures are unavailable in the published English literature.
